# Analysis of ferroptosis-associated genes in Crohn’s disease based on bioinformatics

**DOI:** 10.3389/fmed.2022.1058076

**Published:** 2023-01-13

**Authors:** Xingyu Ji, Su Ma, Xiaomei Sun, Dan Yu, Ye Song, Rui Li

**Affiliations:** ^1^Department of Gastroenterology, The First Affiliated Hospital of Jiamusi University, Jiamusi, China; ^2^Department of Gastroenterology, Heilongjiang Provincial Hospital, Harbin, China; ^3^Department of General Surgery, The First Affiliated Hospital of Harbin Medical University, Harbin, China

**Keywords:** Crohn’s disease, bioinformatics, ferroptosis, Gene Expression Omnibus, inflammatory bowel disease

## Abstract

**Background:**

Ferroptosis, a novel mode of apoptosis has recently been shown to be associated with fibrosis, tumor, cardiovascular, and other diseases. In this study, using bioinformatic analysis, we identified ferroptosis genes associated with Crohn’s disease (CD) and performed biological function analysis, identified potential drug targets, and provided new directions for the future treatment of CD.

**Methods:**

Differential expression analysis was performed using the GSE186582 dataset from the Gene Expression Omnibus (GEO) database. Ferroptosis-associated genes were downloaded from the FerrDB database, and overlapping genes associated with CD and ferroptosis were extracted. Then, we performed functional enrichment analysis, constructed a protein-protein interaction network (PPI), identified the correlation between hub genes and immune infiltration, performed external validation using a second and third dataset (GSE102133, GSE95095), and identified potential therapeutic agents. Finally, we validated the protein expression levels of the identified hub genes by immunohistochemical staining in the colon tissues from CD and healthy participants.

**Results:**

A total of 28 ferroptosis-associated genes associated with CD were identified in our analysis, which included 22 up-regulated and 6 down-regulated genes. Gene Ontology (GO) analysis showed that these genes are essential for the apical plasma membrane and amide transport, and Metascape analysis showed that these genes mainly act on IL-4 and IL-13 signaling pathways. Five hub genes, PTGS2, IL6, IL1B, NOS2, and IDO1, were identified by a protein interaction network, and external validation of these hub genes showed statistically significant differences in expression between the CD patients and normal participants (*p* < 0.05), and all AUC values were greater than 0.8. Further, we predicted the top 10 drugs used to treat CD. Immune infiltration results suggest that Hub gene is related to T cells, macrophages, dendritic cells, and other immune cells. Finally, the results of immunohistochemical experiments showed that the protein expression of the hub gene was higher in CD colon tissue than in normal subjects (*p* < 0.05).

**Conclusion:**

Bioinformatics analysis showed that ferroptosis is closely related to the development of CD, and the prediction of potential drugs provides new targets for the treatment of CD. Moreover, five hub genes identified are potentially new and effective markers for the diagnosis of CD.

## 1. Introduction

Inflammatory bowel disease (IBD) includes two subtypes, namely ulcerative colitis (UC) and Crohn’s disease (CD). CD is a chronic gastrointestinal disease that can affect all segments of the gastrointestinal tract. At the stage of diagnosis, the majority of the patients have manifestations of intestinal inflammation with typical symptoms such as abdominal pain, chronic diarrhea, and weight loss. As the disease progress, fibrosis can occur leading to narrowing of the intestinal lumen, intestinal fistulas, and abscesses. In severe cases of CD surgical treatment may be required, and extraintestinal complications including anemia, osteoporosis, thrombosis, and even cancer might occur (1–3). However, the pathogenesis and etiology of CD are not well defined. Previous studies suggest that cause of CD is a combination of genetic susceptibility, environment, microorganisms, immune damage, and other related factors that cause inflammatory damage (4, 5). The incidence of CD is surging worldwide, with a higher incidence in developed countries than in developing countries, and a higher incidence in urban areas than in rural areas. Moreover, the onset of CD is predominant in adults and adolescent age groups and it is most prevalent in Europe (322/100,000), Canada (319/100,000), and the United States (214/100,000) (6).

Ferroptosis is an iron-dependent, novel form of programmed cell death that is distinct from apoptosis, cellular necrosis, and cellular autophagy. The key factors that trigger ferroptosis are intracellular iron and ROS accumulation, glutathione depletion, and impaired lipid peroxide metabolism (7, 8). Intestinal epithelial cells (IEC) play an important role in the pathogenesis of CD, the intestinal epithelial barrier plays the role of physical barrier and immune barrier to maintain normal intestinal stability and resist bacterial infection. Once the barrier function is damaged, microbes and bacteria in the intestinal cavity may transfer to the intestinal mucosa lamina propria, stimulate immune cells and cytokines, causing immune system disorders in the long term (9). It has been shown that ferroptosis is associated with intestinal epithelial dysfunction, thereby leading to intestinal diseases, such as inflammatory bowel disease, intestinal ischemia/reperfusion injury, and colon cancer (10–12). Studies have shown that excessive intestinal ROS production causes intestinal inflammation in the dextran sodium sulfate (DSS)-induced colitis in mice, an animal model of IBD (13–15).

Currently, there is no research on the mechanism of ferroptosis gene in CD based on bioinformatics. In this research, the public dataset was analyzed, the hub genes were screened out and drug prediction was carried out, to explore the mechanism of this new type of apoptosis in CD. The purpose is to find the genes and pathways related to CD and ferroptosis, and to find new therapeutic drugs, thus providing new ideas for the diagnosis and treatment of CD.

## 2. Materials and methods

### 2.1. Ethical statement

The study was conducted strictly in accordance with the principles of declaration of Helsinki and was approved by the Ethics Committee of Heilongjiang Hospital (Heilongjiang, China).

### 2.2. Data sources

In this study, the dataset GSE186582 was downloaded from the Gene Expression Omnibus (GEO) database to study CD gene expression profile changes. The gene expression profile in this dataset was constructed based on the GPL570 [HG-U133_Plus_2] Affymetrix Human Genome U133 Plus 2.0 Array platform. The dataset contains colon tissue samples from CD patients (*n* = 196), and healthy individuals (*n* = 25).

### 2.3. Sample detection and differential gene analysis

For principal component analysis (PCA), the two R packages FactoMineR and factoextra were used and the LIMMA package was used for differential gene expression analysis. Finally, the genes meeting the criteria such as *p* < 0.05, and | log2 FC| > 1 were included as differentially expressed genes, and the pheatmap package was used for depicting the heat map of the differential genes.

### 2.4. Ferroptosis correlation analysis and Venn analysis

Ferroptosis genes were downloaded from the FerrDband Venn diagrams were plotted using the online tool^1^ to generate Venn diagrams between differentially expressed genes and the ferroptosis-associated genes. Subsequent analyses included differential genes that overlapped with ferroptosis-associated genes. The heat map of the overlapping differentially expressed genes was also plotted using the R software.

### 2.5. Functional enrichment analysis

Gene Ontology (GO) functional enrichment analysis of differential genes associated with ferroptosis was performed using the GO plot package in R. GO analysis included biological process (BP), cellular component (CC), and molecular function (MF). In addition, the obtained differentially expressed genes associated with ferroptosis were subjected to functional enrichment analysis using Metascape, and these genes were uploaded to the Metascape online analysis tool for annotation of their biological processes.

### 2.6. Construction of protein-protein interaction network

Differentially expressed genes associated with ferroptosis were uploaded to the STRINE online database^2^ and analyzed using STRINE’s online database to predict the interaction relationships between proteins encoded by the genes that may play important role in the pathogenesis of CD. Interaction scores > 0.4 were considered significant and their results were downloaded and saved locally for subsequent visualization. The results were visualized using Cytoscape V3.9.0 software and the key top 5 genes were calculated using the cytoHubba plugin.

### 2.7. Correlation analysis of immune infiltration

Based on transcriptome data, single sample gene set enrichment analysis (ssGSEA) algorithm was used to evaluate the immune cell infiltration of patients with Crohn’s disease in GSE186582 dataset. Subsequently, the correlation analysis between the expression levels of 5 core genes and immune cells was carried out. The above analyses were completed by limma package, GSVA package and GSEABase package.

### 2.8. External validation

For validation studies a second dataset GSE102133 constructed based on the GPL6244 [HuGene-1_0-st] Affymetrix Human Gene 1.0 ST Array [transcript (gene) version] platform was downloaded from the GEO database. This dataset contains samples from 65 CD patients and 12 normal human subjects. The expression differences between the two groups were verified, the working characteristic curves (ROC curves) of hub genes were plotted with R to determine the cut-off values and the area under the curve (AUC) was calculated to assess the clinical diagnostic significance of key genes. In addition, GSE95095 was downloaded from GEO database, which contains 48 patients with Crohn’s disease and 12 healthy people, so as to further verify the differential expression of Hub gene between CD and healthy people.

### 2.9. Drug prediction

The Connectivity Map (cMap) database is an online database widely used in pharmacogenomic studies to reveal functional associations of small molecule compounds, genes, and disease states. Correlation scores are obtained based on the enrichment of differentially expressed genes in the reference gene set, with values ranging from −100 to 100, with positive numbers indicating a positive correlation with the reference gene set and negative numbers indicating a negative correlation. In our study, small molecule compounds with correlation scores <−80 were used as promising predicted results.

### 2.10. Analysis of immunohistochemical results

Immunohistochemical staining was performed to validate the differential expression of relevant hub genes in the colon tissues of CD patients and normal subjects. Clinical samples were obtained from archived tissues of 10 patients diagnosed with CD who underwent colonoscopy at the Heilongjiang Provincial Hospital. Similarly, 10 archived colon tissues from healthy participants who underwent colonoscopy. Three diseased and three healthy human tissue specimens were selected for each hub gene. Briefly, five sets of 3-mm paraffin sections were prepared and dried (3V3) and dewaxed using xylene solution and ethanol solution. This was followed by heat induced antigen retrieval and blocking using citrate buffer and endogenous peroxidase, and the slides were then rinsed twice using PBS buffer. IL6 (YT5348,1:200, ImmunoWay), IL1B (YT5201,1:200, ImmunoWay), NOS2 (YT3169,1:200, ImmunoWay), PTGS (YT1073,1:200, ImmunoWay), IDO1 (YN3005,1:200, ImmunoWay) primary antibodies were incubated overnight at 4°C. The sections were then incubated with the secondary antibodies at room temperature for 20 min, washed three times with PBS buffer, and each section was subsequently stained with 3,3′-diaminobenzidine and then re-stained with hematoxylin. Three 200× high magnification fields were randomly observed for each index, and the mean optical density (MOD) values were calculated using Image Pro Plus (IPP) software.

### 2.11. Statistical analysis

The data were analyzed and visualized using R 4.1.0 and SPSS 26.0 statistical software. Differences in hub genes expression in CD and healthy samples were calculated using the *t*-test, and correlation analysis were calculated using Spearman analysis. *P* < 0.05 was considered statistically significant (*p < 0.05, ^**^p < 0.01, ^***^p < 0.001).

## 3. Results

### 3.1. Identification of differentially expressed genes

**FIGURE 1:**
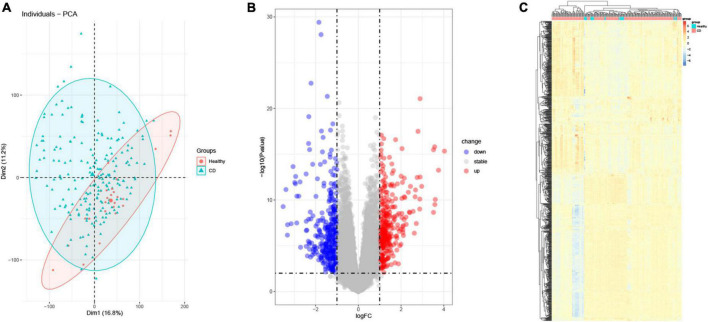
Differential gene analysis (DEG). **(A)** Principal component analysis (PCA). Red represents normal samples, blue represents CD samples. **(B)** Volcano plot differential gene analysis of GSE186582, red represents up-regulated genes and blue represents down-regulated genes. **(C)** Heat map analysis of differential genes.

GSE186582 containing data from 196 CD samples and 25 healthy human samples was downloaded from the GEO database. PCA analysis clearly distinguished the healthy control and CD groups (Figure 1A), and differential expression analysis was performed using the limma package. We found 649 genes differentially expressed (*p* < 0.05 and |log2 FC| > 1) in the colon of CD patients tissues compared with normal human colon tissues, of which 316 genes were up-regulated and 333 genes were down-regulated as shown in the volcano plot (Figure 1B). The differentially expressed genes were clustered using the pheatmap package (Figure 1C).

### 3.2. Ferroptosis-associated genes and Venn analysis

**FIGURE 2:**
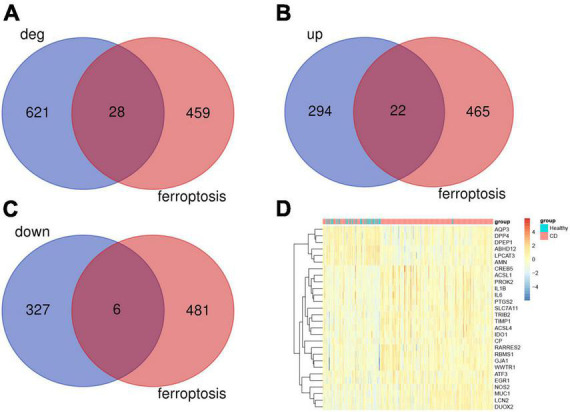
Analysis of ferroptosis-associated genes. **(A)** Ferroptosis gene and Crohn’s disease differential gene Venn analysis, a total of 28 associated differential genes. **(B)** 22 ferroptosis-associated up-regulated genes. **(C)** 6 ferroptosis-associated down-regulated genes. **(D)** Heat map analysis of 28 differentially related genes associated with ferroptosis.

Next, within the differentially expressed genes in the CD disease, we wanted to identify the presence of any ferroptosis-associated genes. Toward this, a list of 487 genes related to ferroptosis was obtained from FerrDb. We then subjected the differentially expressed genes from the GSE186582 dataset and the ferroptosis-associated genes to Venn analysis. We found a total of 28 genes associated with ferroptosis overlapping (Figure 2A), including 22 differentially expressed genes up-regulated in association with ferroptosis (IDO1, NOS2, GJA1, RARRES2, PROK2, ATF3, MUC1, ACSL1, DUOX2, EGR1, WWTR1, PTGS2, IL1B, LCN2 SLC7A11, TIMP1, CP, TRIB2, IL6, ACSL4, RBMS1, CREB5) and 6 down-regulated differential genes (DPP4, AQP3, ABHD12, DPEP1, AMN, LPCAT3) (Figures 2B, C), and these genes were clustered as shown in the heat map (Figure 2D). The Ferroptosis-associated genes were classified into three categories, driver genes (IDO1, GJA1, ATF3, DPP4, AQP3, ACSL1, DUOX2, EGR1, DPEP1, AMN, WWTR1, IL1B, LPCAT3, SLC7A11, TIMP1, IL6, ACSL4), repressor genes (NOS2, RARRES2, PROK2, MUC1, ABHD12, LCN2, SLC7A11, CP, TRIB2, IL6, RBMS1, CREB5), and markers (PTGS2).

### 3.3. Enrichment pathways and analysis of ferroptosis-associated genes

**FIGURE 3:**
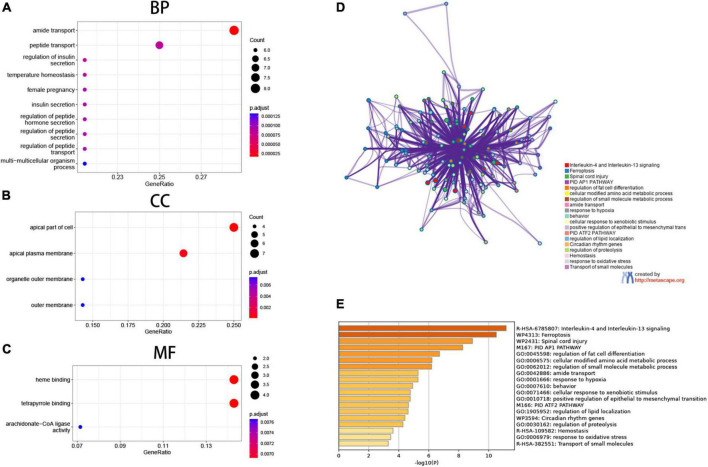
Functional enrichment analysis. **(A–C)** Gene Ontology (GO) enrichment analysis, **(A)** biological process (BP), **(B)** cellular components (CC), **(C)** molecular function (MF). **(D,E)** Matascape gene enrichment analysis IL-1 and IL-13 signaling pathways is most notable.

We then looked at the potential biological functions of these differentially expressed genes that are associated with ferroptosis using R and the online tool Metascape. The most significant enrichment terms analyzed by GO included the apical part of the cell, apical plasma membrane (cellular component); amide transport, (biological process); heme binding, and tetrapyrrole binding (molecular function) (Figures 3A–C). Further, the 28 genes were uploaded to Metascape for online analysis, and as shown in the figure, biological pathways that were significantly enriched included: interleukin-4 and interleukin-13 signaling, ferroptosis, spinal cord injury, and PID AP1 pathway. Also, biological processes such as regulation of fat cell differentiation, cellular modified amino acid metabolic process, and regulation of small molecule metabolic process were significantly enriched (Figures 3D, E).

### 3.4. Protein-protein interaction network analysis of ferroptosis-associated genes

**FIGURE 4:**
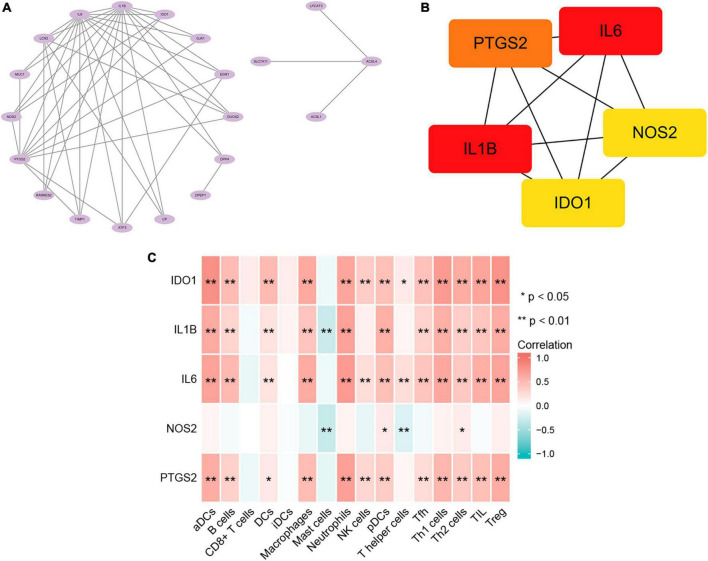
Protein-protein interaction network (PPI) and immune infiltration analysis. **(A)** Visual analysis of ferroptosis-associated genes. **(B)** cytoHubba screening of hub genes. **(C)** Correlation analysis of immune infiltration.

Next, we constructed a network graph using the protein-protein interaction database of the STRING online tool to investigate the role of ferroptosis-associated genes, which contained 28 nodes and 45 edges. In the String database, nodes denote genes, edges denote gene interactions, and the network is set to default as of value (interaction score > 0.4). These 28 genes were analyzed using Cytoscape to construct a network graph, in which 3 genes did not form any network with other genes and were not associated (Figure 4A). The top five genes calculated using a cytoHubba plugin are IL6, IL1B, PTGS2, IDO1, and NOS2 (Figure 4B).

### 3.5. Correlation between hub genes and immune infiltration

In order to further understand the correlation between Hub gene and immune cell infiltration, ssGSEA analysis was carried out. The results showed that the expressions of IDO1, IL1B, IL6, and PTGS2 were positively correlated with the infiltration amount of aDCs, B cells, DCs, Macrophages, Neutrophils, NK cells, pDCs, Tfh, Th1 cells, Th2 cells, TIL, and Treg. NOS2 was negatively correlated with mast cells and T helper cells (Figure 4C). These results further proved that these immune cells played a key role in the progression of Crohn’s disease.

### 3.6. Screening of potential drug targets

Since we narrowed down the list of key genes associated with ferroptosis and CD, we then looked for potential drugs for these targets. To this end, we used cMap for screening potential pharmacological targets, and the drugs were ranked and identified according to their connectivity scores. The results showed that the top ten drugs used for the treatment of CD were: treprostinil, CG-930, mexiletine, ritanserin, LY-2140023, RS-102221, GW-501516, chenodeoxycholic-acid, tyrphostin-AG -1295, neurodazine (Table 1).

**TABLE 1:** Top 10 prediction results from cMap for Crohn’s disease.

Score	Name	Description
−99.93	Treprostinil	Prostacyclin analog
−99.89	CG-930	JNK inhibitor
−99.89	Mexiletine	Sodium channel blocker
−99.86	Ritanserin	Serotonin receptor antagonist
−99.86	LY-2140023	Glutamate receptor agonist
−99.79	RS-102221	Serotonin receptor antagonist
−99.65	GW-501516	PPAR receptor agonist
−99.61	Chenodeoxycholic-acid	11-beta-HSD1 inhibitor
−99.47	Tyrphostin-AG-1295	PDGFR receptor inhibitor
−99.47	Neurodazine	Neurogenesis of non-pluripotent C2C12 myoblast inducer

### 3.7. External validation of hub genes

**FIGURE 5:**
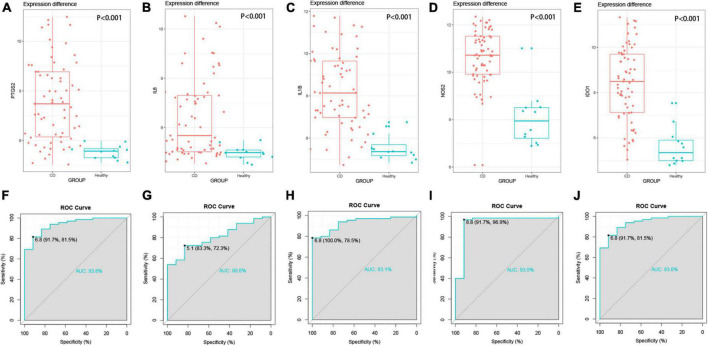
External validation of hub genes. **(A–E)** Hub gene expression between disease group and control group, *P* < 0.001. **(A)** PTGS2, **(B)** IL6, **(C)** IL1B, **(D)** NOS2, **(E)** IDO1, **(F–J)** the ROC curve of the hub gene, the AUC values are all greater than 0.8.

In addition, the results of the previous biological analysis were validated using another CD dataset GSE102133 from the GEO database. We observed that the expression levels of the five genes, PTGS2, IL6, IL1B, NOS2, and IDO1, were significantly higher in the colonic tissues of CD patients compared to the normal subjects (*p* < 0.0001) in the GSE102133 dataset validating our results (Figures 5A–E). To explore the effectiveness of these five genes as potential biological markers for CD, we performed ROC curve analysis on the colon tissues of CD patients and normal subjects. The horizontal and vertical coordinates of the ROC curve indicate sensitivity and specificity, respectively, and a larger AUC indicates a more accurate diagnostic model. The AUC values of PTGS2, IL1B, NOS2, and IDO1 were 0.9, 0.936, 0.931, 0.935, and 0.936, respectively, and the AUC value of IL6 was 0.806. The above results indicated that these five genes had significant differences between CD patients and normal participants and could serve as a good diagnostic biomarker (Figures 5F–J). The expression of hub genes in GSE95095 dataset are statistically significant, and shown in Supplementary Figure 1.

### 3.8. Validation of potential biomarkers using immunohistochemistry

**FIGURE 6:**
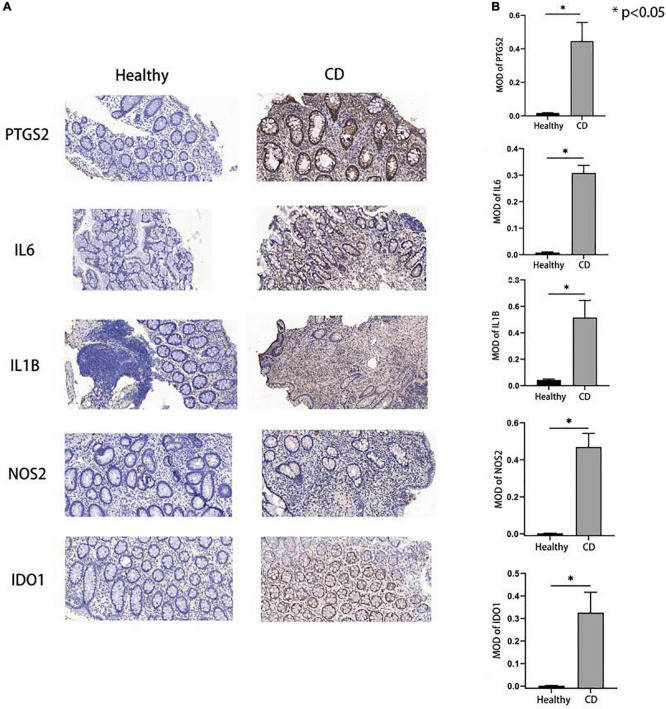
Expression of hub genes in Crohn’s disease and control group. **(A,B)** Immunohistochemical results showed that the hub gene expression in the disease group was higher than that in the control group, and the *p*-values were all less than 0.05, which was statistically significant.

Among the five hub genes identified, IL1B, NOS2, and IDO1 were mainly localized in the cytoplasm; while IL6 and PTGS2 were mainly present in the cell membrane, and brown or golden color represented positive expression. We performed Immunohistochemical analysis in tissues from CD patients and normal participants to validate the expression of these hub genes. Our results showed that the MOD values for the proteins IL6, IL1B, NOS2, PTGS2, and IDO1 were higher in colon tissues of CD patients compared to healthy human colon tissue (all *P*-values were less than 0.05) (Figures 6A, B) indicating a higher protein expression in CD compared to the healthy participants validating our bioinformatic analyses.

## 4. Discussion

In this study, first of all, the differential genes between CD and healthy people is obtained through analysis, subsequently, the differential expression genes related to ferroptosis in CD through screening is obtained, including 22 up-regulated genes and 6 down-regulated genes. PTGS2 is one of the marker genes of ferroptosis, indicating that ferroptosis is involved in the pathogenesis of CD. To figure out the pathogenesis of these genes in CD, functional enrichment analysis has been carried out and it is found that these genes are mainly involved in biological processes including apical plasma membrane and amide transport, and it participates in IL-4 and IL-13 signaling pathways. We also made drug prediction for related genes, and treprotinil, CG-930 and other drugs may become potential drugs for CD treatment. Furthermore, the reliability of bioinformatics analysis is confirmed through the results of external data sets and immunohistochemical validation, providing new ideas for the study of CD pathogenesis and clinical treatment.

Although we did not detect differential expression of GPX4 in our bioinformatics analysis, it is speculated that this might result from the fact that some patients received drug treatment, which affected the results, however, a large number of studies showed that the low expression of GPX4 was closely related to Crohn’s disease. GPX4, a selenoprotein, is a major enzyme that catalyzes the reduction of phospholipid hydrogen peroxide in cells. It can reduce peroxide into corresponding alcohol, inhibit the level of peroxidation in cells, and is of crucial importance to ferroptosis (16, 17). According to Mayr et al., polyunsaturated fatty acids (PUFA) in diet can aggravate Crohn’s disease by disrupting intestinal epithelial oxidative stress reaction. There is low GPX4 activity in tissue samples of CD patients, and the PUFA intake is related to disease activity (18, 19). In addition, under the action of lysophosphatidylcholine acyltransferase 3 (LPCAT3) and acyl coenzyme A synthetase long chain family member 4 (ACSL4), excessive PUFA mediates the generation of phosphatidyl polyunsaturated fatty acids (PUFAPL), resulting in the lipid peroxidation and ferroptosis (18). Moreover, it has been shown in relevant studies that the intestinal glutathione (GSH) of patients with Crohn’s disease are lack of Sido et al.,(20), and GSH is a cofactor of GPX4 antioxidation. GSH deficiency results in GPX4 inactivation, leading to the accumulation of lipid peroxides, as well as ferroptosis. Recent studies have shown that Shaoyao Decoction can reduce TNBS induced colitis by activating Gpx4, inhibiting ferroptosis of intestinal epithelial cells, and restoring barrier function (21).

Gene Ontology and GSEA showed that the differentially expressed genes in CD that were ferroptosis-associated were critical for the apical plasma membrane, amide transport, peptide transport, heme binding, and other biological processes. It was shown that the rate-limiting step of iron absorption in the small intestine is the uptake of iron by the apical cell membrane. Of note, cytochrome b reductase located in the apical membrane of small intestinal cells can reduce Fe3+ to Fe2+, a step that is accelerated when the organism is iron deficient (22). Fe2+ is highly unstable and reactive and generates hydroxyl radicals through the Fenton reaction, thereby reacting directly with the polyunsaturated fatty acids in the cell membrane to generate high lipid ROS, which leads to cell death. About 1/3 of patients with IBD have anemia, 80% of which are iron deficiency anemia (23), which could be explained by the insufficient dietary intake and reduced absorption of iron due to inflammation in the duodenum and jejunum in CD patients (24, 25). However, ferroptosis has also been reported to impair erythropoiesis and disturb systemic iron homeostasis and reticulocyte production which could lead to anemia (26). However, the causal role of ferroptosis if any, in iron deficiency anemia in CD patients and the associated mechanisms need to be investigated in depth. The vast majority of IBD patients take oral and intravenous iron supplements to treat iron deficient anemia, nevertheless, oral iron supplements require prolonged treatment, and suffer from poor absorption, bioavailability, and tolerance hence could aggravate the disease (23, 27). Interestingly, inhibition of ferroptosis-associated genes and balancing iron metabolism are proposed as new treatment options for iron deficiency anemia in CD. Heme-binding protein (HPX) is the protein with the strongest affinity for heme in plasma, and free heme can bind to HPX to form heme-HPX complexes, which are further internalized *via* receptor-mediated endocytosis and catabolized intracellularly (28). Heme oxygenase 1 (HO-1) is a key regulator of cellular ferroptosis and iron metabolism. Under pathological conditions, HO-1 can be upregulated and counteract the toxicity of heme, by converting heme to CO, bilirubin, and Fe2+ (29). Interestingly, in our previous study, intrarectal injection of tranostat improved the symptoms and pathology of DSS-induced colitis mice, potentially through the increased expression of HO-1 by tranostat which induced the expression of the anti-inflammatory factor IL-10 (30, 31). HO-1 acts as a key factor regulating ferroptosis and may potentially regulate IBD as it is highly expressed in IBD. This is consistent with our previous bioinformatics analysis, and therefore we surmise that targeted inhibition of ferroptosis may become a new direction for CD/UC therapy. Functional enrichment using Metascape revealed that differentially expressed genes associated with ferroptosis in CD are mainly involved in the IL-4 and IL-13 signaling pathways. These cytokine pathways are associated with type II helper T lymphocytes (Th cells), a key component of the specific and non-specific immune response (32). It has been reported that IL-4 and IL-13 are involved in the inflammatory response and could promote the switching of the inflammatory phase toward the fibrotic phase by mediating the interaction between T cells and fibroblasts (33). In addition to this, in CD, activation of the STAT6 pathway by IL-4 and IL-13 induces excessive contraction of human intestinal muscle cells, thus promoting the formation of fibrotic strictures in the intestine (34).

We also constructed a protein-protein interaction network using STRING and identified the top 5 hub genes that are related to ferroptosis among the differentially expressed genes in CD by Cytoscape. The top 5 hub genes are PTGS2, IL6, IL-1B, NOS2, and IDO1. PTGS2 is a key gene involved in the activation of ferroptosis-associated lipid peroxidation, acceleration of arachidonic acid metabolism, and promotion of inflammatory signaling molecule secretion (35). PTGS2 also known as COX-2 is a member of cyclooxygenases (COXS) which are a family of oxidoreductases that catalyze the conversion of arachidonic acid to eicosanoids. COX-2 is an inducible isoform that in homeostatic conditions, is expressed at specific stages of the cell cycle and cell differentiation. Further, COX-2 is observed to exhibit differential expression in certain pathological conditions such as inflammation and angiogenesis (36). Interestingly, the COX-2 inhibitor celecoxib was reported to reduce IL-1B levels and decrease colonic injury in a mouse model of experimental enterocolitis (37).

Interleukin-6, another hub gene is an important inflammatory molecule that binds to the interleukin-6 receptor (IL-6R). IL-6R can exist as membrane-bound (mIL-6R) or soluble (sIL-6R), of which sIL- 6R is believed to be the main pathway that triggers signaling. IL-6 and IL-6R can activate the gp130 subunit on the cell surface, which in turn activates related signaling pathways, such as the JAK/STAT pathway (38). In a seminal work, Shikha Nayar et al. found that blocking the common cytokine receptor subunit gp130 ameliorates CD, and proposed gp130 as a therapeutic target for patients who do not respond to anti-TNF therapies and also as a supplemental therapy in addition to the standard treatments for patients with moderate to severe CD (39). There are two main agonists in the IL-1 family, IL-1α and IL-1β, which act as key pro-inflammatory cytokines involved in a variety of autoimmune responses and multiple other cellular activities (40). Dominik Aschenbrenner’s study showed that autocrine and paracrine secretion of IL-1α/IL-1β and IL-10 are the key cytokines controlling IL-23 production by monocytes. Notably, IL-23 signaling and Th1/Th17 immunity are important mechanisms of intestinal inflammation, and thus IL-1α/IL-1β, the upstream of IL-23 inhibition, can be targeted to treat IBD (41). Similarly, Matthias Friedrich et al. also showed that blocking IL-1 may be useful in patients with deep ulcers who have failed to respond to conventional therapies, and hence could potentially improve the outcome of patients with IBD (42). Nitric oxide synthase (NOS) is an isoenzyme with a neural type (NOS1), inducible type (NOS2), and endothelial type (NOS3), which normally presents in endothelial cells, macrophages, neurologic phagocytes, and neuronal cells, respectively. The inducible nitric oxide synthase (NOS2) is expressed after inflammatory injury, but large amounts of NOS2 can produce high concentrations of NO, which cannot be utilized even in the presence of sufficient oxygen, leading to metabolic hypoxia (43). It has also been reported that NOS2 expression is increased in experimental small intestinal ulcers and that the degree of expression correlates with inflammation, and NOS enzyme inhibitors can promote ulcer healing and alleviate inflammation (44). Indoleamine 2,3-dioxygenase (IDO1), which acts on a variety of tryptophan substrates, is the only rate-limiting enzyme other than the liver that catalyzes the catabolism of tryptophan (Trp) within the kynurenine pathway. IDO1 overexpression is shown to deplete tryptophan in the microenvironment, thereby suppressing T-cell function and leading to the immune escape of tumor cells. Recently there have been numerous studies on IDO1 inhibitors for treating tumor-related diseases (45, 46). IDO1, in addition to its role in tryptophan metabolism, a key pathway in intestinal mucosal homeostasis, promotes secretory cell differentiation and mucus secretion in intestinal epithelial cells. Further, IDO1 could interact with AHR and Notch signaling and hence may potentially regulate intestinal epithelial function and promote mucosal healing (47). Of note, mucosal healing is the holy grail of IBD treatment; therefore, upregulation of IDO1 may be a promising direction for the treatment of IBD (47, 48). Our analysis has identified hub genes that have crucial functions in the pathogenesis of CD and could potentially be therapeutic targets for alleviating CD.

In this study, it was found that the infiltration amount of dendritic cells, macrophages, B cells, NK cells, Th1 cells, Th2 cells, and regulatory T cells in patients with Crohn’s disease was increased. After activation, CD4 + T cells differentiated into T cell subsets with different effects, including Th1, Th2, Th17, regulatory T cells (Treg) and follicular helper T cells (Tfh). Th17-mediated immunoregulatory response played an important role in the pathogenesis of CD, and participated in immune response by secreting IL17, IL26, TNF-α, and other cytokines (49). Among them, IL-17 could maintain intestinal homeostasis on macrophages and dendritic cells. IL-10 could promote the expansion of antigen-specific CD24+ and CD25+ regulatory T cells to prevent intestinal hyperinflammation. M1 macrophages generated pro-inflammatory factors such as IL1B, IL6, IL23 and TNF-α, while M2 macrophages secreted anti-inflammatory factors such as IL10 and TGF-β. There were mainly M1 macrophages in IBD patients, which resulted in persistent inflammation (50). Treg could inhibit Th17 activation to maintain immune homeostasis, alleviate intestinal inflammation, and play an important role in negative regulation of immune function. IL-6 could inhibit the differentiation of Treg cells by inducing Th17 differentiation, and plays an important role in regulating the balance of Th17/Treg (51). In addition, the study of Xu et al. showed that the knockout of Gpx4 in Treg cells could increase intracellular lipid peroxidation and ferroptosis levels, and enhance Th17 translation by enhancing IL1B secretion, which affects immune homeostasis (52). These studies indicated that ferroptosis was potentially associated with the pathogenesis of CD, and maintaining the immune balance of patients with Crohn’s disease by inhibiting ferroptosis may be a potential treatment method.

We also identified candidate drug molecules that could target the identified ferroptosis-associated genes. Treprostinil is a stable analog of prostacyclin that promotes direct vasodilation of the systemic arterial vascular bed and inhibits platelet aggregation. Studies have shown that treprostinil activates cAMP, thereby inhibiting tumor growth factor (TGF-β1) secretion and platelet-derived growth factor (PDGF-BB)-induced cell proliferation, and has antifibrotic effects in idiopathic pulmonary fibrosis (IPF) (53). Interestingly, one of the major complications of CD is fibrosis, which leads to excessive collagen production during tissue healing and eventually leads to fibrosis and intestinal stricture. Therefore, Treprostinil may have some promise in the treatment of CD, thus avoiding stenosis due to excessive fibrosis. CG-930 is a JNK inhibitor, and blocking the JNK pathway with a JNK inhibitor has been reported to be effective in alleviating intestinal inflammation in an animal model of IBD (54). In addition, the JNK signaling pathway regulates extracellular matrix production and influences the progression of fibrotic diseases, such as cardiac fibrosis, hepatic fibrosis, renal fibrosis, and pulmonary fibrosis. Given the role of JNK inhibitors in these processes, they are proposed to be an effective treatment for CD fibrosis (55).

There are some limitations to this study. First, the clinical sample size collected in this study was small due to objective reasons such as experimental conditions. Further, the validation study was limited to single-center, small-scale immunohistochemical experiments. Finally, the study of the relevant signaling pathways, hub genes, and potential drug treatment effects in animal models was limited to bioinformatics analysis and lacks further experimental validation.

## 5. Conclusion

In summary, we identified the ferroptosis genes associated with CD using bioinformatics methods and analyzed their pathways, and mechanisms of action to reveal the potential role of ferroptosis in CD and to identify potential therapeutic agents. Our study provides novel targets for the diagnosis and treatment of CD and may be clinically valuable.

## Data availability statement

The datasets presented in this study can be found in online repositories. The names of the repository/repositories and accession number(s) can be found in the article/Supplementary material.

## Ethics statement

The studies involving human participants were reviewed and approved by the Heilongjiang Provincial Hospital Ethics Committee. The patients/participants provided their written informed consent to participate in this study. Written informed consent was obtained from the individual(s) for the publication of any potentially identifiable images or data included in this article.

## Author contributions

XJ and XS designed the experiments. XJ, DY, RL, and SM performed the analysis. XS and DY supervised the study. XJ, YS, and SM wrote the manuscript. All authors contributed to the article and approved the submitted version.

## Footnotes

1
http://bioinformatics.psb.ugent.be/webtools/Venn/

2
https://www.string-db.org/

## Supplementary material

Supplementary Figure 1 Differential expression of hub genes in GSE95095.
