# Supplementary figures and images for: Analysis of ferroptosis-associated genes in Crohn’s disease based on bioinformatics
Source: Front Med (Lausanne). 2023 Jan 13;9:1058076. doi: 10.3389/fmed.2022.1058076 (PMC9881725; doi:10.3389/fmed.2022.1058076)

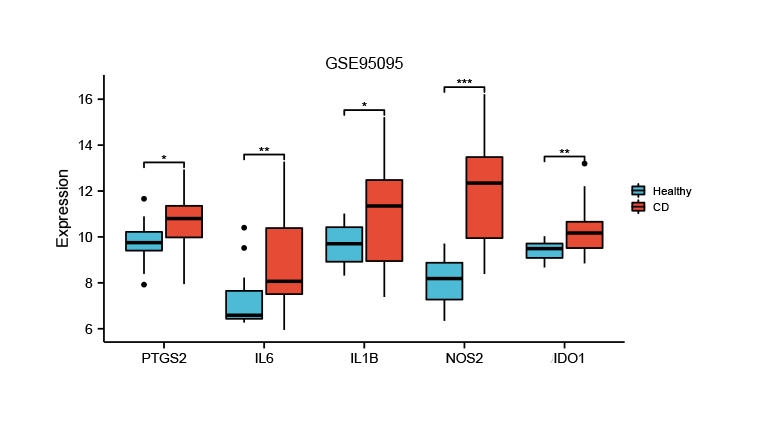

Supplement: Supplementary Figure 1 — Differential expression of hub genes in GSE95095. [file Image_1.TIF]
